# Quercetin Isolated from* Toona sinensis* Leaves Attenuates Hyperglycemia and Protects Hepatocytes in High-Carbohydrate/High-Fat Diet and Alloxan Induced Experimental Diabetic* Mice*

**DOI:** 10.1155/2016/8492780

**Published:** 2016-11-15

**Authors:** Yali Zhang, Huanhuan Dong, Mimi Wang, Jingfang Zhang

**Affiliations:** ^1^Key Laboratory of Biomedical Information Engineering of Ministry of Education, School of Life Science and Technology, Xi'an Jiaotong University, Xi'an 710049, China; ^2^College of Forestry, Northwest A&F University, Yangling 712100, China

## Abstract

The development of diabetes mellitus is related to oxidant stress induced by a high carbohydrate/high-fat diet (HFD). Quercetin, as a major bioactive component in* Toona sinensis* leaves (QTL), is a natural antioxidant. However, the exact mechanism by which QTL ameliorate diabetes mellitus is still unknown. In this study, we investigated the hypoglycemic effects and hepatocytes protection of QTL on HFD and alloxan induced diabetic mice. Intragastric administration of QTL significantly reduced body weight gain, serum glucose, insulin, total cholesterol, triglyceride, low density lipoprotein-cholesterol, alanine aminotransferase, and aspartate aminotransferase serum levels compared to those of diabetic* mice*. Furthermore, it significantly attenuated oxidative stress, as determined by lipid peroxidation, nitric oxide content, and inducible nitric oxide synthase activity and as a result attenuated liver injury. QTL also significantly suppressed the diabetes-induced activation of the p65/NF-*κ*B and ERK1/2/MAPK pathways, as well as caspase-9 and caspase-3 levels in liver tissues of diabetic* mice*. Finally, micrograph analysis of liver samples showed decreased cellular organelle injury in hepatocytes of QTL treated mice. Taken together, QTL can be viewed as a promising dietary agent that can be used to reduce the risk of diabetes mellitus and its secondary complications by ameliorating oxidative stress in the liver.

## 1. Introduction

There is a global increase in the prevalence of diabetes mellitus (DM) which is predominantly related to changing lifestyle and the resulting surge in obesity. DM is a metabolic disease characterized by chronic hyperglycemia resulting from defects in insulin metabolism and impaired function of carbohydrate, lipid, and protein metabolism that leads to long-term complications. The liver plays a central role in whole body lipid and carbohydrate metabolism. The liver dysfunction observed in type 1 and 2 diabetes is described as steatohepatitis, and many authors assign it to a wide group of liver pathologies called nonalcoholic fatty liver disease (NAFLD) [1–3]. Chronic oxidative stress mediated by glucolipotoxicity is involved in the onset and progression of diabetic liver dysfunction in both animal models and humans [4–6]. Hence, regulating liver cell homeostasis and protecting the cells against oxidative surrounding are the most important, which calls for an effective antioxidant therapy as alternative in the management of NAFLD [7–10].

Recently, it has been reported that increased reactive oxygen species (ROS) generation activates stress-sensitive intracellular signaling pathways such as nuclear factor-kappa B (NF-*κ*B), extracellular signal-regulated kinase (ERK), c-Jun N-terminal kinase (JNK), and p38 mitogen-activated protein (MAP) kinase during hepatic dysfunction [11–13]. Several pathological responses have been suggested to induce diabetic complications and these include hyperglycemia, oxidative stress-induced NF-*κ*B transcription, and apoptosis, which all take place synergistically and reciprocally [14–16]. MAPKs participate in apoptotic signal transduction as a response to various factors, including ROS, inflammatory cytokines, ultraviolet lights, radiation, heat, and osmotic shock [17]. Therefore, novel approaches are necessary to identify therapeutic agents which can act with pleiotropic effects including antioxidant properties to prevent and/or treat DM and its secondary complications.

*Toona sinensis* (A. JUSS) M. ROEM belongs to the Mahogany family Meliaceae [18]. The young leaves of* T. sinensis* are used as a vegetable, and mature leaves are used in Chinese traditional medicine for the treatment of enteritis, dysentery, and eye infections. Previous studies revealed that* T. sinensis* leaves are rich in flavonoids with good radical scavenging abilities [19–21]. Importantly, no studies to date have reported significant toxicity of* T. sinensis* leaves. Our previous studies and others have shown that quercetin is the major flavonoid of* T. sinensis* leaves [22, 23]. Pharmacological investigations have demonstrated that quercetin has diverse biological effects such as antioxidant [24], anticancer [25], anti-inflammatory [26], and cardioprotective activities [27, 28]. Quercetin also plays a crucial role in aldose reductase inhibition [29, 30]. Recently, it was found that quercetin has a strong effect on blood glucose levels in alloxan induced hyperglycemia which is mediated by the blunting of free radical induced toxicity [31, 32]. Therefore, quercetin may be one of the main hyperglycemia and dyslipidemia counteracting constituents of* T. sinensis* leaves. However, relatively little attention has been paid to the antihyperglycemic activity of quercetin from* T. sinensis* leaves (QTL), and studies on the effects of QTL in mouse models of diabetes induced by a high-carbohydrate/high-fat diet (HFD) and alloxan have to our best knowledge not been reported to date. Our current study was carried out to determine whether QTL could suppress the hyperglycemia and liver damage induced by HFD-alloxan treatment in diabetic* mice*. The results reveal QTL as a promising dietary agent able to reduce the secondary complications of diabetes mellitus by ameliorating oxidative stress in the liver.

## 2. Materials and Methods

### 2.1. Reagents

Leaves of* T. sinensis* were collected in Shaanxi Province, China, in August 2015 and identified by experts in the College of Forestry, Northwest A&F University, China. Alloxan was purchased from Sigma Chemical Co., USA. Blood glucose (BG) was measured using kits from Shanghai Rongsheng Biotechnology Co., China. Total cholesterol (TC), triglyceride (TG), low density lipoprotein-cholesterol (LDL-C), high density lipoprotein cholesterol (HDL-C), nitric oxide (NO), plasma alanine aminotransferase (ALT), and aspartate aminotransferase (AST) activities were measured using kits from the Nanjing Jiancheng Bioengineering Research Institute, China. Insulin levels were determined using a radioimmunoassay kit from Beijing BioSino Biotechnology Co., China. Polyclonal rabbit antibodies against p65, p38, ERK, caspase-9, and caspase-3 were purchased from Cell Signaling Technologies (Beverly, MA, USA). Antibodies against ß-actin were purchased from Santa Cruz Biotechnology (Santa Cruz, CA, USA). All chemicals were of analytical grade.

### 2.2. Isolation of Quercetin from* T. sinensis *Leaves

*T. sinensis *leaves (1 : 10, w/v) were soaked in a 70% ethanol solvent for 2.5 h and sonicated in an ultrasonic bath at 200 kHz at 55°C for 45 min. Samples were filtered, concentrated, and dried using a rotary evaporator. The dried crude extract was resuspended in distilled water and defatted with petroleum ether. The residue was diluted with H_2_O and extracted using ethyl acetate (EtOAc) in triplicate. Subsequently EtOAc fractions were chromatographed over silica gel (n-hexane/EtOAc/MeOH) to obtain 13 fractions. Fraction 8, eluted with EtOAc/MeOH (8 : 1), was further separated and purified by capillary electrophoresis using silica gel column chromatography to yield quercetin. The structure of isolate was determined by reverse phase high performance liquid chromatography in comparison with authentic quercetin (the National Institute for the Control of Pharmaceutical and Biological Products, Beijing, China).

### 2.3. Animals, Diets, and Experimental Design

Male Chinese Kunming mice weighing 20 ± 2 g (*n* = 60) were obtained from the Experimental Animal Center of Xi'an Jiaotong University (Xi'an, China) and were acclimated for 1 week before being randomly assigned to different experimental groups. The mice were maintained on a 12 h light/dark cycle on a standard chow diet until experimental analysis. The experimental animal protocol was approved by the Experimental Animal Ethics Committee of Xi'an Jiaotong University.

After adaptation for 1 week, the mice were randomly divided into four groups (*n* = 15 per group, 5 mice per cage): (1) normal; (2) normal + 200 mg/kg b.w./d QTL; (3) DM groups: high-carbohydrate/high-fat diet- (HFD-) alloxan treatment (HFD, 52.6% standard laboratory chow, 10% lard, 15% sucrose, 15% yolk powder, 5% casein, 1.2% cholesterol, 0.2% bile salt, 0.6% calcium bicarbonate, and 4.73 kcal/gram); (4) DM + 200 mg/kg b.w./d QTL. After 4 weeks of dietary manipulation, mice fed with HFD were injected intraperitoneally with 0.04% alloxan dissolved in sterile normal saline in a dose of 100 mg/kg b.w. The mice were allowed to continue to feed on their respective diets until the termination of the experiment. Water and food were available ad libitum. Body weight and food intake were recorded weekly. Three weeks after alloxan injection, the animals were sacrificed by euthanization with isofluorane after fasting for 8 h; plasma and liver were collected, weighed, shock frozen in liquid nitrogen, and stored at −80°C for further analysis.

### 2.4. Biochemical Analysis of Blood Samples

Blood samples were collected by cardiac puncture, and plasma was obtained by centrifuging the blood at 8000 ×g for 15 min at 4°C. Fasting blood glucose levels were monitored periodically with the tail prick method using kits based on glucose oxidase method. Blood glucose levels were expressed as mmol/L.

For the oral glucose tolerance test, mice were fasted for 8 hours but given water freely and either left untreated or treated with QTL 90 minutes before the test was performed using D-glucose at a dose of 2 g/kg b.w. Blood was drawn from the tail vein before treatment and at 0, 30, and 120 minutes after the glycemic load.

The levels of insulin, TC, TG, HDL-C, and LDL-C, as well as the activities of ALT and AST in sera, were measured according to the commercial kits instructions.

### 2.5. Liver Oxidative Stress Levels

Livers were thawed, weighed, and homogenized in buffer (5 mM Tris-HCl, 2 mM EDTA, pH 7.4) on ice. Homogenates were centrifuged (150 ×g, 10 min, 4°C) and the supernatants were used immediately for the assays. NO content and iNOS activity were measured according to the test kits instructions.

### 2.6. Western Blot Protein Assay

Whole protein lysates of liver tissue were extracted using RIPA buffer (25 mM Tris-HCl (pH 7.6), 150 mM NaCl, 1% NP-40, 1% sodium deoxycholate, and 0.1% SDS, Thermo Fisher Scientific) supplemented with 1% protease inhibitor cocktail and 1% phenylmethylsulfonyl fluoride. Protein concentrations were measured using a BCA Protein Assay Kit. Aliquots containing 50 *μ*g of protein were loaded onto an 8% SDS-PAGE gel, transblotted onto a polyvinylidene difluoride (PVDF) membrane (Bio-Rad Laboratories, USA), blocked for 1 h at room temperature with 5% bovine serum albumin in TBS buffer, and subsequently incubated overnight with respective primary antibodies against p65, p38, ERK, caspase-9, caspase-3, and ß-actin (internal control). The membrane was then incubated with secondary HRP conjugated antibodies (ZB 2301, Zhongshan Golden Bridge Biotechnology Co. Ltd., China). The bound complexes were detected with a chemiluminescence solution (ECL) purchased from Millipore (#WBKLS0050, Merck Millipore, USA) according to the manufacturer's instructions. Chemiluminescence was imaged on a LAS-3000 system (FUJIHILM, Tokyo, Japan). The immunoblot bands were quantified using densitometric analysis, and the ratio of each protein to ß-actin was calculated and normalized to the values of the mice fed the normal chow (set as 1).

### 2.7. Histological Observations of Liver

Slices of liver tissue were fixed in 10% formalin for 1 week at room temperature. The specimens were subsequently dehydrated in a graded series of ethanol solutions, cleared in xylene, and embedded in paraffin wax. Tissue blocks were sectioned into sections of 5 *μ*m thickness using a rotary microtome and stained with hematoxylin and eosin (H&E). Histological changes in the stained sections were inspected under a light microscope by a pathologist without knowledge of the experimental groups the samples belonged to.

### 2.8. Transmission Electron Microscopy

Portion of liver (about 1 mm^3^) from the control and experimental groups of mice was fixed in 3% glutaraldehyde in sodium phosphate buffer (200 mM, pH 7.4) for 3 h at 4°C. Tissue samples were washed with the same buffer and additionally fixed in 1% osmium tetroxide and in sodium phosphate buffer (200 mM, pH 7.4) for 1 h at 4°C. The samples were washed again with the same buffer for 3 h at 4°C, dehydrated with a graded series of ethanol solutions, and embedded in araldite. Thin sections were cut using a LKBUM4 ultramicrotome equipped with a diamond knife (Diatome, Aldermaston, Berkshire, England), mounted on a copper grid, and stained with 2% uranyl acetate and Reynolds lead citrate. The grids were examined under a Hitachi H-7650 transmission electron microscope (Hitachi High-Technologies Corporation, Japan).

### 2.9. Statistical Analysis

Means and standard deviations were used for statistical analysis. Significance of differences between means was evaluated using one-way analysis of variance (ANOVA); frequency data were compared using Ridit scoring. A difference was considered significant at *P* < 0.05.

## 3. Results

### 3.1. Hyperglycemic Effect of QTL

**Figure 1:**
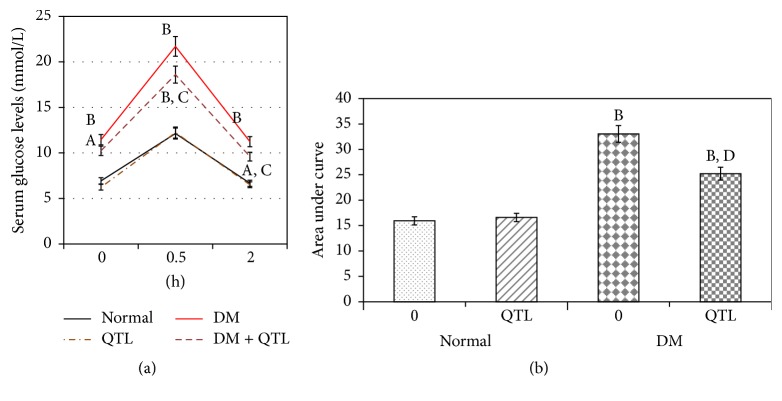
Effects of QTL on oral glucose tolerance (a) and AUC (b) in normal and diabetic* mice*. Data represent the mean ± SD from ten animals in each group. ^A^ *P* < 0.05 compared to control group; ^B^ *P* < 0.01 compared to control group; ^C^ *P* < 0.05 compared to diabetic group; ^D^ *P* < 0.01 compared to diabetic group.

Table 1 presents the changes in body weight, blood glucose levels, and plasma insulin levels in normal and diabetic mice treated with QTL. QTL treatment did not affect weight gain, blood glucose levels, and insulin levels of normal mice; however it significantly lowered the weight gain (*P* < 0.01), blood glucose levels (*P* < 0.01), and insulin levels (*P* < 0.01) within the diabetic groups (Table 1). As shown in Figures 1(a) and 1(b), the glucose tolerance and AUC (area under curve) of the diabetic control mice were significantly worse than those of normal controls (*P* < 0.05, *P* < 0.01, resp.), and both the diabetic and control groups treated with QTL showed a significant improvement (*P* < 0.05, *P* < 0.01, resp.) in their tolerance performance.

### 3.2. Effect of QTL on Serum Lipid Metabolic Parameters

Table 2 shows the effect of QTL on lipid metabolic parameters. At termination, TG, TC, and LDL-C levels in the diabetic groups were significantly (*P* < 0.01) higher than those from normal controls, indicating a disorder of lipid metabolism in the untreated diabetic groups (Table 2). QTL treatment in the diabetic groups significantly (*P* < 0.01) decreased TG and TC levels. Additionally, HDL-C levels were significantly (*P* < 0.01) lower in diabetic groups compared to those of the normal diet and normal diet supplemented with QTL groups. Even although QTL treatment did not result in significant downregulation of LDL-C levels (*P* > 0.05) in the diabetic mice (Table 2), it significantly (*P* < 0.01) improved HDL-C levels in this group.

### 3.3. Effects of QTL on Oxidative Stress and Hepatic Function Markers in the Serum

**Figure 2:**
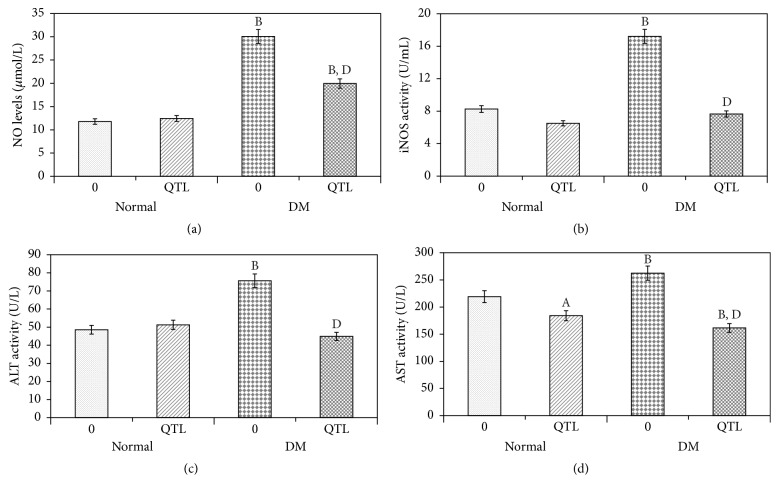
Effects of QTL on oxidative stress and hepatic function markers in serum of normal and diabetic* mice*. NO level (a); iNOS activity (b); ALT activity (c); and AST activity (d). Data represent the mean ± SD from ten animals in each group. ^A^ *P* < 0.05 compared to control group; ^B^ *P* < 0.01 compared to control group; ^C^ *P* < 0.05 compared to diabetic group; ^D^ *P* < 0.01 compared to diabetic group.

As shown in Figures 2(a) and 2(b), QTL had no notable effect on the serum NO levels and iNOS activity of normal mice. However, HFD-alloxan induced diabetic mice showed significantly (*P* < 0.01) enhanced hepatic NO levels and iNOS activity, and QTL treatment led to a significant restoration of NO levels and iNOS activity towards normal values (*P* < 0.01, *P* < 0.01, resp.) when compared with nontreated diabetic mice (Figures 2(a) and 2(b)).

ALT and AST activity in diabetic mice was significantly higher (*P* < 0.01, *P* < 0.01, resp.) than that in normal mice and normal mice treated with QTL (Figures 2(c) and 2(d)). After 42 d of oral QTL administration, the ALT and AST activity in the treated diabetic mice was lower than that in the untreated diabetic mice, and no significant difference (*P* > 0.05) was found for ALT activity between normal mice and QTL treated diabetic mice. The higher levels of ALT observed in the diabetic mice could indicate a certain degree of hepatic damage, and these results consequently indicated that QTL protects against hepatic injury induced in type 2 diabetic mice.

### 3.4. Histopathological Observations

**Figure 3:**
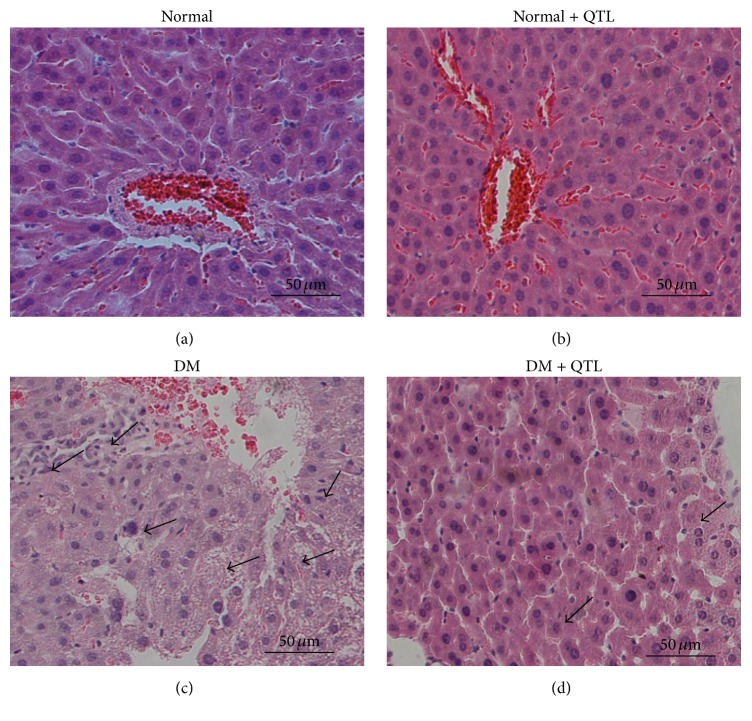
Effect of QTL on hepatic morphological changes (H&E staining). (a) Normal; (b) normal + QTL; (c) diabetic; (d) diabetic + QTL; (*n* = 3; 20x; scale bar 100 *μ*m).

In the control animals, a normal liver architecture was observed with a normal central vein and dense cytoplasm with no granulocytes. In the diabetes model group, the liver showed marked alterations as evidenced by extensive cell necrosis, congestion, karyolysis, infiltration, and inflammation (Figure 3). Administration of QTL to diabetic mice ameliorated these deleterious effects in a dose-dependent manner.

### 3.5. Histopathological TEM Observations

**Figure 4:**
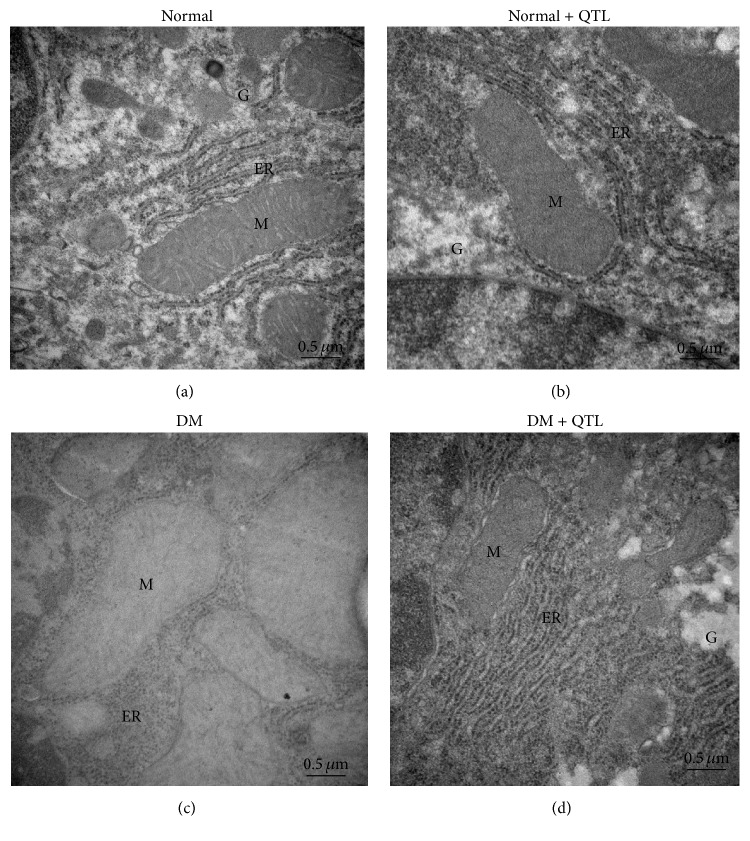
Transmission electron microscopy of hepatocytes of normal and diabetic mice. At the end of experimental period, the mice were sacrificed and the hepatic tissues were sectioned for the transmission electron microscopic studies. Transmission electron micrographs of (a) normal, (b) normal + QTL, (c) diabetic, and (d) diabetic + QTL treated mice's hepatic tissue sections were showed at 10,000x magnification. Endoplasmic reticulum (ER), glycogen (G), and mitochondria (M).

The ultrastructural changes observed in the hepatocytes of normal and diabetic mice are shown in Figures 4(a)–4(d).  Figure 4(a) is representative of the electron micrographs of hepatocytes of normal mice showing normal cellular organelles, mitochondria, and rough endoplasmic reticulum, as well as a nucleus with intact nuclear membrane and normal chromatin. Similar architecture is observed in the electron micrographs of normal mice treated with QTL (Figure 4(b)). The electron micrographs of hepatocyte of diabetic mice (Figure 4(c)) revealed the decrease of organelle regeneration, swelling in the cisternae of the rough endoplasmic reticulum and mitochondrial cristae, fusion or disappearance of mitochondrial crests, degranulation of rough endoplasmic reticulum, increased smooth endoplasmic reticulum and lipid accumulation, and cells with dark and light cytoplasm. The electron micrograph of cells from treated diabetic animals (Figure 4(d)) shows the protective effect of QTL on hepatocyte in diabetic mice, as illustrated by either absent or significantly reduced swelling of the rough endoplasmic reticulum cisternae and mitochondrial cristae, reduction of smooth endoplasmic reticulum, and accumulation of parallel rough endoplasmic reticulum cisternae dotted with ribosomes.

### 3.6. Hepatic Expression of Apoptosis-Related Proteins

**Figure 5:**
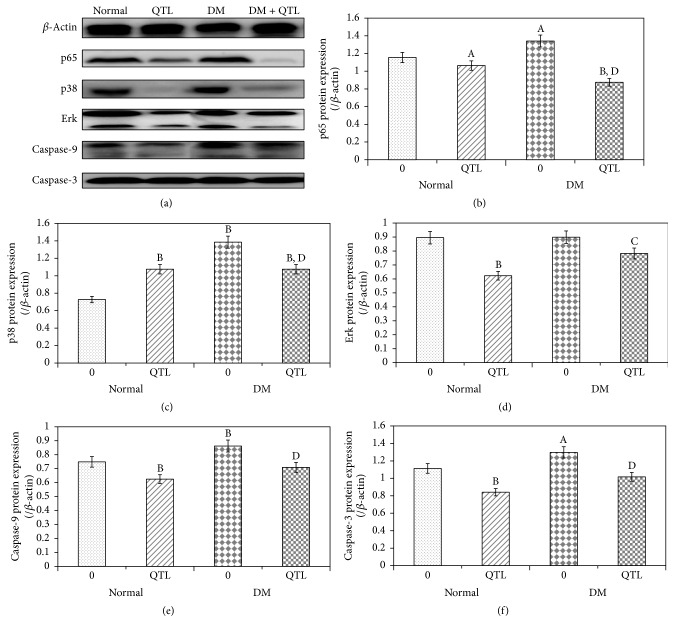
p65, p38, ERK, caspase-9, and caspase-3 proteins expression analysis in the liver of normal and diabetic* mice*. Density values were normalized to *β*-actin levels. Data represent the mean ± SD from three animals in each group. ^A^ *P* < 0.05 compared to control group; ^B^ *P* < 0.01 compared to control group; ^C^ *P* < 0.05 compared to diabetic group; ^D^ *P* < 0.01 compared to diabetic group.

The expression levels of p65, p38, ERK, caspase-9, and caspase-3 proteins in the liver were analyzed (Figure 5). A clear increase in NF-*κ*B/p65 protein levels was produced in the diabetic mice (*P* < 0.01), induced by treatment with HFD-alloxan. Western blotting and the subsequent measurement of band intensity relative to *β*-actin indicated that treatment with QTL markedly (*P* < 0.01) inhibited HFD-alloxan induced NF-*κ*B/p65 activation. The expression of p38 and ERK was significantly increased (*P* < 0.01 and *P* < 0.01, resp.) in diabetic mice compared to normal mice, whereas only ERK enhanced levels were significantly decreased by QTL administration (*P* < 0.01). Caspase-9 and caspase-3 protein expressions were also higher in diabetic mice than normal mice, and again the expression levels of markers were significantly decreased in the QTL treated group (*P* < 0.01 and *P* < 0.01, resp.).

## 4. Discussion

Over the years, various medicinal plants and their extracts rich in polyphenolic/flavonoid compounds have been reported to be effective against reactive oxygen species mediated damage by enhancing antioxidants defenses and reducing hyperglycemia in alloxan or streptozotocin-induced diabetes. In the present study, we investigated the possible usefulness of the potent antioxidant quercetin isolated from* T. sinensis* leaves in a mouse diabetes model induced by HFD-alloxan treatment.

Previous studies showed that 0.12 g/kg b.w and 0.06 g/kg b.w. of total flavonoid from* T. sinensis* could significantly decrease the blood glucose levels in mice with alloxan induced diabetes [19]. A study by Cao et al. demonstrated that 100 mg/kg b.w quercetin ameliorated intracellular stress, led to better regulated gene expression, and reduced embryonic malformations in a diabetic pregnancy model [33]. Jeong et al. fed a diet containing 0.04% quercetin (equivalent to 60~120 mg/kg b.w.) or 0.08% of the diet (equivalent to 120~240 mg/kg b.w.) to C57BL/KsJ-db/db mice for 6 weeks. The results showed quercetin to be effective in improving hyperglycemia, dyslipidemia, and antioxidant status in type 2 diabetes [32]. Zahedi et al. evaluated the effects of quercetin intake on cardiovascular risk factors and inflammatory biomarkers in 72 women with type 2 diabetes in a double-blind randomized clinical trial. Quercetin was given to participants as a 500 mg capsule daily. The results found that quercetin supplementation reduced systolic blood pressure significantly but had no effect on other cardiovascular risk factors and inflammatory biomarkers [34]. Considering the biological effects of quercetin, we chose 200 mg/kg b.w. QTL for the present study. Daily oral treatment of normal mice with QTL (200 mg/kg b.w.) for 49 d led only to nonsignificant differences in body weight gain, blood glucose, insulin, TC, HDL-C levels, ALT activity, NO levels, and iNOS activity. QTL significantly reduced the TG, LDL-C levels, and AST activity in the sera of normal mice. Furthermore, QTL induced a significant decline in the protein levels of bax, caspase-9, and caspase-3 in livers of normal mice. These results indicated that QTL has no toxic effects on normal mice.

Quercetin was reported to possess *α*-glucosidase inhibitory activity in vitro [35] as well as a hyperglycemia ameliorating activity in vivo which most likely exerts by blunting free radical induced toxicity in type 2 diabetes mellitus [31]. In the present study, administration of QTL to diabetic mice led to a significant decrease in body weight gain and fasting blood glucose, as well as an increase in serum insulin levels. Metabolic disturbances and their consequences in diabetes mellitus are well known, and hyperlipidemia is one of a set of the disorders characteristics of diabetes. Some studies also showed that quercetin can significantly decrease blood cholesterol levels in rats fed with a high cholesterol diet that induces lipemic-oxidative injury, leading the authors to consider it as an effective hypocholesterolemic agent [36]. We have also assessed the serum levels of TC, TG, LDL-C, and HDL-C, and QTL treatment that resulted in a significant recovery in the levels of these lipid profile biosensors. This shows that QTL has the ability to lower the level of LDL cholesterol while at the same time boosting the HDL cholesterol level.

It has been reported that diabetic mice had significantly increased levels of iNOS expression and activity in the serum [37, 38]. Excess NO produced by iNOS has been reported to induce deleterious effects in the livers of diabetic mice [38]. Our data showed that treatment with QTL caused a marked reduction in NO production and iNOS activity in the serum. These parameters showed that QTL suppressed the oxidative stress caused by HFD-alloxan induced diabetes.

Epidemiological studies show that diabetic patients are at higher risk of developing chronic liver diseases and hepatocellular carcinoma. In experimental models of diabetes, both a high-carbohydrate/high-fat diet and alloxan exert their toxic effects on the liver and other organs in addition to pancreatic *β*-cells. The insulin insufficiency and hyperglycemia that result from *β*-cell necrosis further augment liver damage through reactive free radical mediated lipid peroxidation of hepatocellular membrane [39, 40]. In this study, the diabetic hyperglycemia induced by HFD-alloxan treatment produced elevated levels of AST and ALT in the serum, which are considered highly indicative of liver dysfunction. QTL treatment normalized the AST and ALT levels in diabetic mice. These results, together with the observed histological changes, indicated that QTL protects against hepatic injury in diabetic mice.

Accumulating evidence indicates that hyperglycemia induced oxidative stress leads to peripheral tissue injury in diabetic patients [41–43]. Oxidative stress is associated with the modulation of NF-*κ*B, MAPK, and caspase dependent pathways in experimental model of diabetic liver injury [44–46]. NF-*κ*B is one of the most ubiquitous transcription factors and regulates the expression of genes required for cellular proliferation, the inflammatory response, and cell adhesion. The expression of iNOS is also regulated by NF-*κ*B, which is ubiquitous in the cytoplasm as a p50 and p65 heterodimer [47, 48]. MAPKs, with their three subgroups, regulate various physiological and pathological cellular action [49]. JNK and p38 MAPK are identified as “stress activated kinases” that are generally related to inflammation and apoptosis. ERK supports cellular proliferation by responding to epidermal growth factors (EGFs) and other extrinsic mitogenic signals [49]. In this study, HFD-alloxan treatment was able to activate the NF-*κ*B and MAPK pathways in mouse hepatocytes of mice, whereas the expressions of p65 and ERK proteins were markedly reduced by QTL administration. Those results suggest that QTL protect hepatic damage through extrinsic mitogenic signals.

The caspase cascade is the most prevalent pathway for apoptotic signal transduction in liver cells [50]. Activation of caspase-3 is a key step in both the extrinsic and intrinsic apoptotic pathways and is often considered to be the point of no return in the apoptotic signaling cascade. Interestingly, the present study revealed that QTL treatment of diabetic mice significantly suppressed hepatic caspase-9 and caspase-3 protein expression. The results presented here suggest that QTL could prevent apoptosis-induced hepatic damage, at least in part, through the amelioration of oxidative stress-induced diabetic liver injury.

## 5. Conclusion

The present study shows that quercetin from* Toona sinensis* leaves exhibits significant antihyperglycemic and liver cell protective effects in a high-carbohydrate/high-fat diet and alloxan induced mouse model of diabetes. The observed liver cell protection by quercetin may involve inhibition of pathways related to oxidative stress. These results will be crucial to understand the role* T. sinensis* leaves may play as alternative or supplementary therapies for clinical treatment of diabetes.

## Figures and Tables

**Table 1:** Effect of QTL on body weight gain, glucose, and insulin levels in the normal and diabetic mice.

Group	Body weight gain (g)	Blood glucose levels (mmol/L)	Plasma insulin levels (*μ*U/mL)
Normal	6.18 ± 0.18	6.93 ± 0.87	39.25 ± 3.25
Normal + QTL	6.13 ± 0.52	6.23 ± 0.31	39.02 ± 2.46
DM	9.73 ± 2.41^B^	13.45 ± 2.16^B^	70.31 ± 1.19^B^
DM + QTL	7.39 ± 0.96^B,D^	7.59 ± 0.68^B,D^	30.11 ± 3.65^D^

Values were expressed as mean ± SD, *n* = 10 for each group. ^A^ *P* < 0.05 compared to control group; ^B^ *P* < 0.01 compared to control group; ^C^ *P* < 0.05 compared to diabetic group; ^D^ *P* < 0.01 compared to diabetic group.

**Table 2:** Effect of QTL on lipid metabolic parameters in the normal and diabetic *mice*.

Group	Total cholesterol (mmol/gprot)	Triglyceride (mmol/gprot)	LDL-C (mmol/L)	HDL-C (mmol/L)	HDL/LDL
Normal	3.73 ± 1.24	0.47 ± 0.24	0.42 ± 0.09	1.22 ± 0.23	2.9
Normal + QTL	2.84 ± 1.95^B^	0.45 ± 0.92	0.39 ± 0.14^B^	1.43 ± 0.21	3.7^B^
DM	6.72 ± 0.83^B^	0.85 ± 0.18^B^	0.69 ± 0.13	1.05 ± 0.19^B^	1.5^B^
DM + QTL	4.15 ± 1.16^A,D^	0.58 ± 0.35^A,D^	0.45 ± 0.10^B^	2.19 ± 0.38^B,D^	4.9^B,D^

Values were expressed as mean ± SD, *n* = 7 for each group. ^A^ *P* < 0.05 compared to control group. ^B^ *P* < 0.01 compared to control group. ^C^ *P* < 0.05 compared to diabetic group. ^D^ *P* < 0.01 compared to diabetic group.

## Abbreviations

QTL: Quercetin from* Toona sinensis* leaves
DM: Diabetes mellitus
HFD: High-carbohydrate/high-fat diet
TC: Total cholesterol
TG: Triglyceride
LDL-C: Low density lipoprotein-cholesterol
HDL-C: High density lipoprotein-cholesterol
ALT: Alanine aminotransferase
AST: Aspartate aminotransferase
NO: Nitric oxide
iNOS: Inducible nitric oxide synthase
RT-PCR: Reverse transcription polymerase chain reaction
ROS: Reactive oxygen species.

## References

[1] Williams K. H., Burns K., Constantino M. (2015). An association of large-fibre peripheral nerve dysfunction with non-invasive measures of liver fibrosis secondary to non-alcoholic fatty liver disease in diabetes. *Journal of Diabetes and Its Complications*.

[2] Litao M. K., El-Baba M. F., Buggs-Saxton C. (2016). Hepatomegaly and liver dysfunction in a 15-year-old girl with type 1 diabetes mellitus. *Clinical Pediatrics*.

[3] Bonapace S., Perseghin G., Molon G. (2012). Nonalcoholic fatty liver disease is associated with left ventricular diastolic dysfunction in patients with type 2 diabetes. *Diabetes Care*.

[4] Bullon P., Newman H. N., Battino M. (2014). Obesity, diabetes mellitus, atherosclerosis and chronic periodontitis: a shared pathology via oxidative stress and mitochondrial dysfunction?. *Periodontology 2000*.

[5] Crujeiras A. B., Díaz-Lagares A., Carreira M. C., Amil M., Casanueva F. F. (2013). Oxidative stress associated to dysfunctional adipose tissue: a potential link between obesity, type 2 diabetes mellitus and breast cancer. *Free Radical Research*.

[6] Stefanović A., Kotur-Stevuljević J., Spasić S., Bogavac-Stanojević N., Bujisić N. (2008). The influence of obesity on the oxidative stress status and the concentration of leptin in type 2 diabetes mellitus patients. *Diabetes Research and Clinical Practice*.

[7] Musso G., Anty R., Petta S. (2013). Antioxidant therapy and drugs interfering with lipid metabolism: could they be effective in NAFLD patients?. *Current Pharmaceutical Design*.

[8] Shams M. E. E., Al-Gayyar M. M. H., Barakat E. A. M. E. (2011). Type 2 diabetes mellitus-induced hyperglycemia in patients with NAFLD and normal LFTs: relationship to lipid profile, oxidative stress and pro-inflammatory cytokines. *Scientia Pharmaceutica*.

[9] Du D., Shi Y.-H., Le G.-W. (2010). Oxidative stress induced by high-glucose diet in liver of C57BL/6J mice and its underlying mechanism. *Molecular Biology Reports*.

[10] Raffaella C., Francesca B., Italia F., Marina P., Giovanna L., Susanna I. (2008). Alterations in hepatic mitochondrial compartment in a model of obesity and insulin resistance. *Obesity*.

[11] Feng B., Ruiz M. A., Chakrabarti S. (2013). Oxidative-stress-induced epigenetic changes in chronic diabetic complications. *Canadian Journal of Physiology and Pharmacology*.

[12] Kujiraoka T., Satoh Y., Ayaori M. (2013). Hepatic extracellular signal-regulated kinase 2 suppresses endoplasmic reticulum stress and protects from oxidative stress and endothelial dysfunction. *Journal of the American Heart Association*.

[13] Manna P., Das J., Ghosh J., Sil P. C. (2010). Contribution of type 1 diabetes to rat liver dysfunction and cellular damage via activation of NOS, PARP, I*κ*B*α*/NF-*κ*B, MAPKs, and mitochondria-dependent pathways: prophylactic role of arjunolic acid. *Free Radical Biology and Medicine*.

[14] Zhong L., Luo Y., Huang C., Liu L. (2011). Effect of NF-*κ*B decoy on insulin resistance of adipocytes from patients with type 2 diabetes mellitus. *Diabetes and Metabolism*.

[15] Zhao Y., Krishnamurthy B., Mollah Z. U. A., Kay T. W. H., Thomas H. E. (2011). NF-*κ*B in type 1 diabetes. *Inflammation & Allergy-Drug Targets*.

[16] Granic I., Dolga A. M., Nijholt I. M., Van Dijk G., Eisel U. L. M. (2009). Inflammation and NF-*κ*B in Alzheimer's disease and diabetes. *Journal of Alzheimer's Disease*.

[17] Thandavarayan R. A., Giridharan V. V., Sari F. R. (2011). Depletion of 14-3-3 protein exacerbates cardiac oxidative stress, inflammation and remodeling process via modulation of MAPK/NF-*κ*B signaling pathways after streptozotocin-induced diabetes mellitus. *Cellular Physiology and Biochemistry*.

[18] Hsieh T.-J., Liu T.-Z., Chia Y.-C. (2004). Protective effect of methyl gallate from Toona sinensis (Meliaceae) against hydrogen peroxide-induced oxidative stress and DNA damage in MDCK cells. *Food and Chemical Toxicology*.

[19] Zhang J.-F., Yang J.-Y., Wen J., Wang D.-Y., Yang M., Liu Q.-Q. (2008). Experimental studies on hypoglycemic effects of total flavonoid from Toona sinensis. *Zhong Yao Cai*.

[20] Hseu Y.-C., Chang W.-H., Chen C.-S. (2008). Antioxidant activities of Toona Sinensis leaves extracts using different antioxidant models. *Food and Chemical Toxicology*.

[21] Yang H., Gu Q., Gao T. (2014). Flavonols and derivatives of gallic acid from young leaves of *Toona sinensis* (A. Juss.) Roemer and evaluation of their anti-oxidant capacity by chemical methods. *Pharmacognosy Magazine*.

[22] Yu W.-J., Chang C.-C., Kuo T.-F., Tsai T.-C., Chang S.-J. (2012). Toona sinensis Roem leaf extracts improve antioxidant activity in the liver of rats under oxidative stress. *Food and Chemical Toxicology*.

[23] Chao P.-Y., Lin S.-Y., Lin K.-H. (2014). Antioxidant activity in extracts of 27 indigenous taiwanese vegetables. *Nutrients*.

[24] Molina M. F., Sanchez-Reus I., Iglesias I., Benedi J. (2003). Quercetin, a flavonoid antioxidant, prevents and protects against ethanol-induced oxidative stress in mouse liver. *Biological and Pharmaceutical Bulletin*.

[25] Shankar G. M., Antony J., Anto R. J. (2015). Quercetin and tryptanthrin: two broad spectrum anticancer agents for future chemotherapeutic interventions. *Enzymes*.

[26] Hisanaga A., Mukai R., Sakao K., Terao J., Hou D. (2016). Back cover: anti-inflammatory effects and molecular mechanisms of 8-prenyl quercetin. *Molecular Nutrition & Food Research*.

[27] Daubney J., Bonner P. L., Hargreaves A. J., Dickenson J. M. (2015). Cardioprotective and cardiotoxic effects of quercetin and two of its in vivo metabolites on differentiated H9c2 cardiomyocytes. *Basic and Clinical Pharmacology and Toxicology*.

[28] Chen Y.-W., Chou H.-C., Lin S.-T. (2013). Cardioprotective effects of quercetin in cardiomyocyte under ischemia/reperfusion injury. *Evidence-Based Complementary and Alternative Medicine*.

[29] Milackova I., Prnova M. S., Majekova M. (2015). 2-Chloro-1,4-naphthoquinone derivative of quercetin as an inhibitor of aldose reductase and anti-inflammatory agent. *Journal of Enzyme Inhibition and Medicinal Chemistry*.

[30] Enomoto S., Okada Y., Güvenc A., Erdurak C. S., Coşkun M., Okuyama T. (2004). Inhibitory effect of traditional Turkish folk medicines on aldose reductase (AR) and hematological activity, and on AR inhibitory activity of quercetin-3-*O*-methyl ether isolated from *Cistus laurifolius* L.. *Biological and Pharmaceutical Bulletin*.

[31] Alam M. M., Meerza D., Naseem I. (2014). Protective effect of quercetin on hyperglycemia, oxidative stress and DNA damage in alloxan induced type 2 diabetic mice. *Life Sciences*.

[32] Jeong S.-M., Kang M.-J., Choi H.-N., Kim J.-H., Kim J.-I. (2012). Quercetin ameliorates hyperglycemia and dyslipidemia and improves antioxidant status in type 2 diabetic db/db mice. *Nutrition Research and Practice*.

[33] Cao L., Tan C., Meng F., Liu P., Reece E. A., Zhao Z. (2016). Amelioration of intracellular stress and reduction of neural tube defects in embryos of diabetic mice by phytochemical quercetin. *Scientific Reports*.

[34] Zahedi M., Ghiasvand R., Feizi A., Asgari G., Darvish L. (2013). Does quercetin improve cardiovascular risk factors and inflammatory biomarkers in women with type 2 diabetes: a double-blind randomized controlled clinical trial. *International Journal of Preventive Medicine*.

[35] Li Y. Q., Zhou F. C., Gao F., Bian J. S., Shan F. (2009). Comparative evaluation of quercetin, isoquercetin and rutin as inhibitors of *α*-glucosidase. *Journal of Agricultural and Food Chemistry*.

[36] Mariee A. D., Abd-Allah G. M., El-Beshbishy H. A. (2012). Protective effect of dietary flavonoid quercetin against lipemic-oxidative hepatic injury in hypercholesterolemic rats. *Pharmaceutical Biology*.

[37] Yang J. Y., Kang M. Y., Nam S. H., Friedman M. (2012). Antidiabetic effects of rice hull smoke extract in alloxan-induced diabetic mice. *Journal of Agricultural and Food Chemistry*.

[38] Fujimoto M., Shimizu N., Kunii K., Martyn J. A. J., Ueki K., Kaneki M. (2005). A role for iNOS in fasting hyperglycemia and impaired insulin signaling in the liver of obese diabetic mice. *Diabetes*.

[39] Alevizos I., Misra J., Bullen J. (2007). Linking hepatic transcriptional changes to high-fat diet induced physiology for diabetes-prone and obese-resistant mice. *Cell Cycle*.

[40] Feng W., Zhao T., Mao G. (2015). Type 2 diabetic rats on diet supplemented with chromium malate show improved glycometabolism, glycometabolism-related enzyme levels and lipid metabolism. *PLoS ONE*.

[41] Karasu Ç. (2010). Glycoxidative stress and cardiovascular complications in experimentally-induced diabetes: effects of antioxidant treatment. *Open Cardiovascular Medicine Journal*.

[42] Esper R., Vilariño J., Machado R., Paragano A. (2008). Endothelial dysfunction in normal and abnormal glucose metabolism. *Advances in Cardiology*.

[43] Uyanik M. H., Albayrak A., Odabasoglu F. (2012). Effects of diabetes on cytokines and oxidative organ injury in a rat model of sepsis. *Cellular and Molecular Biology*.

[44] Soetikno V., Sari F. R., Sukumaran V. (2012). Curcumin prevents diabetic cardiomyopathy in streptozotocin-induced diabetic rats: possible involvement of PKC-MAPK signaling pathway. *European Journal of Pharmaceutical Sciences*.

[45] Cellek S., Qu W., Schmidt A. M., Moncada S. (2004). Synergistic action of advanced glycation and products and endogenous nitric oxide leads to neuronal apoptosis in vitro: a new insight into selective nitrergic neuropathy in diabetes. *Diabetologia*.

[46] Cacicedo J. M., Benjachareowong S., Chou E., Ruderman N. B., Ido Y. (2005). Palmitate-induced apoptosis in cultured bovine retinal pericytes: roles of NAD(P)H oxidase, oxidant stress, and ceramide. *Diabetes*.

[47] Kleinert H., Pautz A., Linker K., Schwarz P. M. (2004). Regulation of the expression of inducible nitric oxide synthase. *European Journal of Pharmacology*.

[48] Brands M. W., Manhiani M. M. (2012). Sodium-retaining effect of insulin in diabetes. *American Journal of Physiology—Regulatory, Integrative and Comparative Physiology*.

[49] Kim E. K., Choi E.-J. (2015). Compromised MAPK signaling in human diseases: an update. *Archives of Toxicology*.

[50] Tak P. P., Gerlag D. M., Aupperle K. R. (2001). Inhibitor of nuclear factor *κ*B kinase *β* is a key regulator of synovial inflammation. *Arthritis & Rheumatism*.

